# Associations between community-based physiotherapy for musculoskeletal injury and health related quality of life (EQ-5D): a multi-centre retrospective analysis

**DOI:** 10.1186/s12955-017-0789-3

**Published:** 2017-10-25

**Authors:** Nick Caplan, H. Robson, A. Robson, G. Barry, G. Wilkes

**Affiliations:** 1 Faculty of Health and Life Sciences, Northumbria University, Northumberland Building, Newcastle upon Tyne, NE1 8ST UK; 2 Connect Health, Newcastle upon Tyne, UK

**Keywords:** Rehabilitation, Intervention, Physical therapy, Quality of life, Musculoskeletal

## Abstract

**Background:**

Community-based musculoskeletal physiotherapy is used to improve function and health related quality of life (HRQoL). The purpose of this retrospective, multi-centre observational study was to determine the association between community-based physiotherapy management for musculoskeletal disorders and changes in HRQoL.

**Methods:**

Four thousand one hundred twelve patients’ data were included in the study. Patients were included if they received a single period of treatment for a musculoskeletal injury or disorder. Patients were only included if they were being treated for a single morbidity. Patients received standard physiotherapy appropriate to their specific disorder, which could include health education/advice, exercise therapy, manual therapy, taping, soft tissue techniques, electrotherapy and/or acupuncture. Health related quality of life was assessed using the EQ-5D index.

**Results:**

EQ-5D improved by 0.203 across all patients (d = 1.10). When grouped by anatomical site of symptom, the largest increases in EQ-5D was in foot pain (0.233; d = 1.29) and lumbar pain (0.231; d = 1.13). Improvements in EQ-5D greater than the minimum clinically important difference (MCID) were seen in 68.4% of all patients. The highest proportion of patients with positive responses to treatment were in ankle pain (74.2%) and thoracic pain (73.4%). The hand (40.5%), elbow (34.7%), and hip (33.9%) showed the greatest proportion of patients that did not respond to treatment.

**Conclusions:**

Community-based musculoskeletal physiotherapy is associated with improved health related quality of life. A randomised controlled trial is needed to determine any causal relationship between community-based physiotherapy and health related quality of life improvements.

## Background

Musculoskeletal injuries and disorders are one of the largest contributors to pain and disability worldwide [1]. Low back pain is often reported as the most common site of musculoskeletal pain with prevalence reported between 25% [2] to 39% [3], followed by the neck, knee and shoulder [2]. Musculoskeletal pain of at least moderate intensity has been reported in approximately one fifth of the European population [4], and can lead to significant national healthcare costs and associated costs to the economy through absence from work [5]. Musculoskeletal pain and disability also lead to reductions in health-related quality of life (HRQoL) in comparison to the general population. As well as treating the musculoskeletal disorder, physiotherapists aim to improve patients’ HRQoL [6].

The EuroQol 5 Dimensions (EQ-5D) instrument provides an indication of general health status across five dimensions including mobility, self-care, usual activities, pain/discomfort, and anxiety/depression [7]. It also expresses an overall estimate of health status by calculating an index based on the results from each of the five dimensions in comparison to national benchmarks. In the United Kingdom, this index ranges from −0.594-1, where 1 is the best possible quality of life and below zero indicates quality of life worse than death [8]. The EQ-5D has been used in population surveys [9–11] and across a range of musculoskeletal conditions [12–19]. It has also been used to evaluate the effectiveness of orthopaedic surgical interventions on HRQoL [20].

In physiotherapy, EQ-5D has been used to assess the effectiveness of interventions such as the PhysioDirect telephone service [21], interventions for back and neck pain [13, 17], acupuncture [14, 15], postural exercise [16], aquatic exercise therapy [22], and advanced physiotherapy services such as a musculoskeletal clinical assessment and treatment service (CATS) [23] and corticosteroidal injections [18, 19]. It is the preferred HRQoL outcome recommended by the Chartered Society of Physiotherapy [24] and the National Institute for Clinical Excellence [25] in the United Kingdom. To the best of our knowledge, however, no studies have yet evaluated changes in HRQoL following community-based physiotherapy interventions across a wide range of musculoskeletal injuries/disorders, and there are also no comprehensive sources of normative EQ-5D data for musculoskeletal patients. The aim of this study was, therefore, to determine the association of community-based physiotherapy treatment for musculoskeletal pain with changes in HRQoL, and to determine whether these associations are influenced by the anatomical site of pain.

## Methods

### Population

All patients that received National Health Service (NHS) community-based physiotherapy from one of five centres (South West Essex, Camden, Gateshead, Newcastle, Northumberland) between January 2012 and April 2016 were included in the sample and data collected before and after their episode of care were assessed retrospectively. The mean new to review ratio across these centres within this period was 1:2.38. Patients entered the service either via referral from their general practitioner (GP), or through self-referral via “Physioline”, a telephone assessment and advice service. Those aged less than 16 years of age, or those with hearing impairments were excluded from the telephone self-referral system and entered only through GP referral.

### Ethical approval statement

The study was approved by the Northumbria University Ethics Committee as a retrospective service evaluation.

### Intervention

On first entry into the service, patients’ anatomical site of pain was coded into one of 13 categories, including the foot, ankle, knee, hip, sacroiliac, lumbar, thoracic, neck, temporomandibular, shoulder, elbow, wrist, hand, as well as “generalised pain” and “other disorders not otherwise stated”. Patients subsequently received physiotherapy-based management in a community setting appropriate to their disorder or injury. These interventions typically included exercise therapy, manual therapy, taping, soft tissue techniques, electrotherapy and/or acupuncture, and were chosen according to patient need, using shared decision making. Episodes of care usually involved an initial telephone triage and treatment assessment followed by face-to-face appointments, if required.

### Outcomes

Patients entering the service completed a pre-intervention EQ-5D. As only the English language version of the instrument was used, those unable to understand the English language sufficiently to answer the questions without the need for an interpreter did not complete a pre- or post-intervention EQ-5D and were excluded from this study.

The EQ-5D was administered upon first entry into the physiotherapy service and requested upon discharge from the service following the completion of treatment. Within each of the five dimensions, a patient will receive an integer score from 1 to 5, where a score of 1 indicates the best outcome (full health) for that dimension. From the individual dimension scores, an index is calculated according to national tariffs. As this study evaluated EQ-5D data from a UK population, the UK tariff was used to calculate the EQ-5D index [8], which can range from −0.594-1.0.

Previous studies have presented the minimum clinically important difference (MCID) in EQ-5D index in musculoskeletal patients, including a MCID of 0.03 [26] and 0.081 [27] in low back pain, 0.08 in total hip arthroplasty patients [28], 0.121 in knee osteoarthritis and 0.054 in patients undergoing limb reconstruction [27]. Jansson and Granath [20] assessed changes in EQ-5D index following orthopaedic surgery, sub-grouping patients into positive responders (EQ-5D index increased by >0.1) and negative responders (EQ-5D decreased by >0.1). For the current study, a change in EQ-5D of >0.1 was used to indicate responders, as used previously by Jansson and Granath [20], providing a relatively conservative treatment of the data in comparison to the lower MCIDs reported previously [26–28].

### Data handling

The investigators had full access to the data from all five centres, which were recorded in a single nationwide system. Data were only included for patients that were being treated for a single morbidity, where the length of time in the service was between 2 and 16 weeks. This was to ensure that only data for a single treatment intervention were included, being deemed the minimum and maximum duration that a patient would remain in the service for a single bout of treatment. Given a study period of 4 years, some patients re-entered the service at a later date for a further assessment and treatment, and these were included in the study as separate records. Any duplicate records were removed prior to analysis. Patients were only included if their records showed both pre- and post-treatment EQ-5D index. Patients were excluded if they were referred to advanced community services (CATS), as those patients had their post-treatment EQ-5D within CATS which, therefore, did not make this a valid representation of community-based physiotherapy management. Sacroiliac pain, temporomandibular pain and general pain groups were removed from the dataset due to having very low patient numbers, and the other disorders not otherwise stated group was also removed as it did not pertain to any specific anatomical location.

### Data analysis

Demographic and EQ-5D data were evaluated for all patients, and for individual sites of pain. The number of patients of each gender were determined overall, as were the number of patients that received treatment within each site of pain. Age, days in service and EQ-5D data were reported as means (±SD) for all patients, by gender, and by site of pain. Changes in EQ-5D index between the start and end of treatment within the physiotherapy service were analysed using paired samples t tests for each site of pain. A 95% confidence level was used for all statistical tests. All statistical comparisons were performed in IBM SPSS version 24. As observed in our data, as well as in previous studies, EQ-5D indices are often skewed towards a score of 1.0 [20], suggesting that the data were not normally distributed. Despite this, due to the large sample size, the data can be treated as parametric in accordance with the central limit theorem [29]. Cohen’s d effect size was used to determine the magnitude of effect of any change in EQ-5D in order to provide a relative change between pre- and post-treatment based on the magnitude of change and the standardised variance between datasets. Effect size was classified as trivial where d < 0.2, small where 0.2 < d < 0.5, moderate where 0.5 < d < 0.8, and large where d > 0.8.

## Results

Overall, 33,117 patient records were obtained. After removal of duplicates (*n* = 4832), 28,285 patients had a first EQ-5D recorded, of whom 5547 patients also had an end of episode EQ-5D recorded, who were included in the analysis. Those without a follow-up score (*n* = 22,275) were either discharged after their telephone assessment only as a “one-stop shop”, or failed to return the EQ-5D score after discharge if seen face to face. There were 465 patients that had returned more than 2 EQ-5D scores, who were excluded from the analysis due to the data management system only retaining the first and last score. Of the 5547 patients with both a pre- and post-treatment EQ-5D, a further 1275 were excluded as they did not meet the remaining inclusion criteria. Due to very small patient numbers, those in the sacroiliac pain (*n* = 24), temporomandibular pain (*n* = 4), and generalised pain (n = 4) were excluded, as were patients classified as other disorder not otherwise stated (*n* = 128), leaving a final sample for analysis of 4112 patient records.

The mean age of patients overall was 50.8 (±17.4) years, and the mean time to complete treatment was 50 (±25) days (Table 1). More female patients (59.5%) received physiotherapy treatment than males (40.5%), although the mean age was similar between females (51.2 ± 17.6 years) and males (50.4 ± 17.2 years). Male patients received treatment for an average of 4 days less than female patients.

**Table 1:** Patient demographics overall, by gender and by site of pain

			Age		Female	Days in service	
	N	%	mean	SD	%	mean	SD
All patients	4112	100.0	50.8	17.4	59.5	50	25
Excluded patients	28,285	–	48.8	17.3	61.7	–	–
Gender							
Female	2447	59.5	51.2	17.6	–	51	26
Male	1664	40.5	50.4	17.2	–	47	25
Site of pain							
*Lower limb*							
Foot pain	107	2.6	50.5	15.6	68.2	43.3	23.0
Ankle pain	190	4.6	44.7	17.8	61.1	50.2	27.0
Knee pain	791	19.3	51.5	18.5	60.2	48.8	24.4
Hip pain	301	7.3	56.8	17.8	68.8	50.8	25.1
*Trunk and head*							
Lumbar pain	991	24.2	49.8	16.7	57.9	48.6	25.8
Thoracic pain	128	3.1	42.1	17.6	57.0	48.7	26.5
Neck pain	563	13.7	50.9	17.0	65.4	50.5	25.3
*Upper limb*							
Shoulder pain	706	17.2	53.4	16.9	51.3	52.8	26.1
Elbow pain	150	3.7	48.6	12.7	54.0	50.2	24.9
Wrist pain	88	2.1	44.0	17.2	59.1	46.1	24.1
Hand pain	84	2.0	52.8	18.5	65.5	43.2	23.7

The most common sites of pain reported included lumbar (24.2%), knee (19.3%), shoulder (17.2%) and neck (13.7%) pain. The least common sites of pain included hand (2.0%), wrist (2.1%), and foot (2.6%) pain.

Overall, the EQ-5D index improved significantly by 0.203 (t(4111) = 62.453, *p* < 0.001; CI = 0.197–0.203), with a large effect size (d = 1.10) (Table 2). Female patients improved by 0.203 (t(2447) = 46.828, p < 0.001; CI = 0.195–0.203; d = 1.10) and male patients by 0.202 (t(1663) = 41.469, p < 0.001; CI = 0.193–0.203; d = 1.12). The majority (68.1%) of patients were classed as responders in terms of their EQ-5D index increasing by at least 0.1, with 31.9% being reported as non-responders. Male patients showed a smaller proportion of non-responders (31.2%) compared to female patients (32.4%). A significant increase in EQ-5D was associated with community-based physiotherapy management in patients with foot (t(106) = 10.991, *p* < 0.001; CI = 0.191–0.233; d = 1.29), ankle (t(189) = 14.871, *p* < 0.001; CI = 0.183–0.211; d = 1.36), knee (t(790) = 28.882, *p* < 0.001; CI = 0.187–0.200; d = 1.14), hip (t(300) = 15.587, *p* < 0.001; CI = 0.159–0.182; d = 1.08), lumbar (t(990) = 30.004, *p* < 0.001; CI = 0.216–0.231; d = 1.13), thoracic (t(127) = 14.496, *p* < 0.001; CI = 0.147–0.170; d = 1.28), neck (t(562) = 22.968, p < 0.001; CI = 0.179–0.196; d = 1.09), shoulder (t(705) = 25.904, p < 0.001; CI = 0.185–0.200; d = 1.08), elbow (t(149) = 12.201, p < 0.001; CI = 0.144–0.172; d = 1.08), wrist (t(87) = 10.419, p < 0.001; CI = 0.141–0.174; d = 1.25) and hand (t(83) = 8.389, p < 0.001; CI = 0.122–0.160; d = 0.88) pain.. The largest proportion of positive responders was found in patients being treated for ankle (74.2%) and thoracic (73.4%) pain. The highest proportion of patients not showing a clinically meaningful increase in EQ-5D was found in patients being treated for hand (40.5%), elbow (34.7%), and hip (33.9%) pain.

**Table 2:** EQ-5D data overall, by gender and by site of pain

	EQ-5D index score at baseline		EQ-5D index score post rehab		EQ-5D change from baseline			EQ-5D responders		EQ-5D non-responders	
	mean	SD	mean	SD	mean	*p*	d	n	%	n	%
All patients	0.648	0.207	0.851	0.161	0.203	<0.0001	1.10	2800	68.1	1312.0	31.9
Excluded patients	0.638	0.241	–	–	–	–	–	–	–	–	–
Gender											
Female	0.640	0.208	0.843	0.164	0.203	<0.0001	1.10	1654	67.6	793.0	32.4
Male	0.661	0.205	0.864	0.157	0.203	<0.0001	1.12	1145	68.8	519.0	31.2
Site of pain											
*Lower limb*											
Foot pain	0.614	0.216	0.847	0.145	0.233	<0.0001	1.29	75	70.1	32.0	29.9
Ankle pain	0.674	0.177	0.885	0.133	0.211	<0.0001	1.36	141	74.2	49.0	25.8
Knee pain	0.654	0.190	0.855	0.161	0.200	<0.0001	1.14	538	68.0	253.0	32.0
Hip pain	0.657	0.183	0.838	0.155	0.182	<0.0001	1.08	188	62.5	113.0	37.5
*Trunk and head*											
Lumbar pain	0.603	0.233	0.834	0.177	0.231	<0.0001	1.13	690	69.6	301.0	30.4
Thoracic pain	0.728	0.138	0.898	0.127	0.170	<0.0001	1.28	94	73.4	34.0	26.6
Neck pain	0.660	0.201	0.856	0.159	0.196	<0.0001	1.09	382	67.9	181.0	32.1
*Upper limb*											
Shoulder pain	0.656	0.214	0.856	0.156	0.200	<0.0001	1.08	482	68.3	224.0	31.7
Elbow pain	0.694	0.175	0.866	0.144	0.172	<0.0001	1.08	94	62.7	56.0	37.3
Wrist pain	0.693	0.148	0.867	0.130	0.174	<0.0001	1.25	62	70.5	26.0	29.5
Hand pain	0.695	0.222	0.855	0.141	0.160	<0.0001	0.88	47	56.0	37.0	44.0

## Discussion

The main findings of this study were that attending community-based physiotherapy for musculoskeletal injury was associated with a significant increase in EQ-5D, both overall, and in all sites of pain. Two thirds of patients overall and by gender reported a clinically meaningful increase in EQ-5D following physiotherapy management. Between 56.0–81.3% of patients showed clinically meaningful increases (>0.1) in EQ-5D index across all sites of pain, and less than 5% of patients showed reductions in EQ-5D overall, by gender, and across all sites of pain.

Overall, EQ-5D improved significantly by 0.203 across all sites of pain over the intervention period, with a large magnitude of effect. We believe this finding may provide new evidence that face -to-face community-based physiotherapy interventions are efficacious across a range of musculoskeletal presentations in improving health-related quality of life. Given that 68.4% of patients reported a clinically meaningful increase in EQ-5D, this should provide confidence for investment in physiotherapy as a modality in any musculoskeletal healthcare pathway. However, this suggestion must be confirmed in a prospective randomised controlled trial, as changes in EQ-5D reported in the retrospective cohort used in the present study could also have been the result of improvement due to the natural course of the condition over time.

Only a limited number of studies have previously explored the associations between musculoskeletal-specific interventions and EQ-5D, reporting improvements ranging from 0.04 to 0.31 across physiotherapy, advanced physiotherapy, and inpatient musculoskeletal care [13–19, 22, 23, 30]. Typically, those patients with the greatest improvement had the lowest baseline index score [14], and those showing the smallest improvement had the highest baseline index [16]. In comparison to the majority of studies with similar baseline indices, patients in the current study showed, on average, greater improvements from baseline to end of episode [13, 17–19], and approximately double the improvement seen previously in patients with back and neck pain that received standard physiotherapy treatment [13, 17]. These studies all used prospective patient cohorts. The fact that the patient cohort in the current study reported larger improvements in EQ-5D gives some confidence that the improvements in HRQoL could be due, at least in part, to the physiotherapy management. A randomised controlled trial is warranted to confirm any causal relationship, and to ensure that the changes in EQ-5D observed were not simply the result of the natural healing process.

The most commonly treated sites of pain were the lumbar spine (24.2%), knee (19.3%), shoulder (17.2%) and neck (13.7%). In a large sample of patients with chronic pain, Parsons et al. [2] reported low back pain in 25%, neck pain in 18%, knee pain in 17% and shoulder pain in 17% of patients, which compares well to the current sample. A higher proportion of patients in their study had neck pain than observed here, which could be a result of the inclusion criteria used in the present study to exclude patients with co-morbidities. Low back pain was more common in female patients, which supports previous literature [3].

At baseline, EQ-5D was 0.648 across all patients, and was higher in male patients (0.661) than female patients (0.640). The lowest pre-treatment EQ-5D was found in patients with lumbar pain (0.603) and was highest in patients with thoracic pain (0.728). When grouped by site of pain, the largest improvement was seen in patients treated for foot (0.233) and lumbar (0.231) pain, and the smallest change was seen in patients receiving treatment for hand (0.160), although this still showed a large effect magnitude.

Compared with a range of orthopaedic surgical interventions where EQ-5D was reported to increase by a mean of 0.18 [20], our cohort of physiotherapy managed patients had favourable outcomes. It should be noted that hip and knee arthroplasty specifically were shown to be more successful in improving HRQoL than our physiotherapy service outcomes. It appears that by the time knee and hip osteoarthritic patients receive an arthroplasty, their level of pain and disability is far worse than many of the knee and hip patients in the current study, as is suggested by the reduced baseline EQ-5D in knee and hip arthroplasty patients [20] compared to the more general knee and hip physiotherapy population. For patients with elbow or hand disorders, patients were least likely to show a positive response to treatment (change in EQ-5D >0.1), which was also found following elbow/hand surgery [20].

The EQ-5D provides an overall indication of health status which is independent of the disorder for which patients are receiving treatment. Some studies have suggested that it could be more relevant to develop disease specific outcome measures, rather than using generic HRQoL outcomes. For example, the STarT Back screening tool was developed to subgroup low back pain patients for targeted treatment [31]. Whilst disease specific outcomes could be a useful adjunct to assessing HRQoL, on their own they may not be sensitive to poor general health outcomes despite a positive response on local joint function. Generic HRQoL outcome measures will still provide useful information for health economic analyses used by commissioners [32].

A limitation of this study was that it was a retrospective analysis of data collected as part of routine clinical practice. As such, it was only possible to determine whether there was an associated change in HRQoL over the time course of physiotherapy management, as discussed above. The time period between EQ-5D scores also differed between patients as the outcome was administered on first entry into the service and on successful discharge. As the service took a patient-centred approach to determining the most appropriate course of treatment, and due to patients presenting at varying stages of injury/dysfunction, such variance in treatment length was to be expected. A prospective randomised controlled trial is needed to determine whether there is a causal relationship between community-based physiotherapy for musculoskeletal disorder and improvements in HRQoL or whether these changes were due to the natural healing process.

This was however, the largest reported sample of musculoskeletal patients with EQ-5D index both pre- and post-treatment to date, and used data from a typical population of patients receiving community-based physiotherapy management for musculoskeletal injury. For those patients that self-referred through the Physioline service, the pre-treatment EQ-5D was administered over the telephone rather than in a face-to-face consultation for GP referred patients. McPhail et al. [33] showed high reliability between EQ-5D completed over the telephone and face-to-face, supporting the validity of the current data. As this was a multi-centre study, a large number of physiotherapists would have delivered interventions to the patients included. Whilst this could increase the variability of the treatments received, standard clinical guidelines were used across all five centres.

Another potential limitation of this study is that a large number of patients failed to complete a second EQ-5D, and so had to be excluded, which could lead to selection bias. Completing second scores involves patient (e.g. patient did not attend treatment) and clinician factors (e.g. clinician did not administer post-treatment EQ-5D) and is a common challenge in clinical practice. The outcomes of those not completing are unknown and open to speculation. However, given the size of the sample, we believe this study gives the best indication of the association between physiotherapy interventions and improvements in HRQoL. Baseline data for the excluded patients (Tables 1 and 2) show that the excluded patients were a similar age to the included patients, with a similar gender split and similar baseline EQ-5D scores, suggesting that selection bias was likely to be very small. However, the current study did exclude 78.8% of patients that, at least, entered the service due to them not having a post-treatment EQ-5D. Completion of both pre- and post-treatment EQ-5D should, therefore, be considered essential for community-based physiotherapy services to more comprehensively evaluate their impact on HRQoL.

## Conclusions

In conclusion, there was an association between community-based physiotherapy and improvements in HRQoL in all joint areas. Community-based physiotherapy management was associated with the highest proportion of patients reporting a clinically meaningful increased in EQ-5D in ankle pain and thoracic pain, and with the smallest proportion reporting clinically meaningful increases in EQ-5D in hand, elbow and hip pain. A randomized controlled trial is needed in order to determine any causal relationship of the associations observed between community-based physiotherapy for musculoskeletal disorder and improvements in HRQoL.
